# Influence of Regular Video Game Play on the Basic Skills of Arthroscopic Surgery – Results of a Prospective Study

**DOI:** 10.1007/s40670-026-02827-z

**Published:** 2026-07-20

**Authors:** Tom Folkerts, Lorena Günter, Georg Gosheger, Carolin Rickert, Dennis Liem

**Affiliations:** 1 Center for Musculoskeletal Surgery, Charité, University Hospital Berlin, Berlin, Germany; 2 Department of General Orthopaedics and Tumororthopaedics, University Hospital Muenster, Muenster, Germany; 3 Sporthopaedicum Berlin, Berlin, Germany

**Keywords:** Surgical training, Arthroscopy, Medical education, Video games, Skills, Teaching

## Abstract

**Introduction:**

The increasing demand for arthroscopic procedures, combined with limited access to affordable training opportunities, emphasizes the need to explore alternative methods for developing essential surgical skills. The objective of this study was to determine whether regular video game playing, known to enhance visuospatial perception and motor skills, improves initial performance on an arthroscopic box trainer and to investigate the relationship between video game proficiency and basic arthroscopic skills.

**Methods:**

This prospective study included 50 students. Participants were divided into two groups, gamers and non-gamers, based on their weekly gaming activity. Arthroscopic skills were assessed using three specific modules on an arthroscopic box trainer, while video gaming performance was assessed using Super Monkey Ball (Nintendo Wii) and Splatoon 2 (Nintendo Switch). Quantitative analyses were performed for all tasks, including time and error rates.

**Results:**

Fifty participants (76% male, 24% female; age 19–30 years) were analyzed. Gamers performed significantly better in Splatoon (*p* < 0.001), while differences in Super Monkey Ball were not significant (*p* = 0.172). In the arthroscopy modules, completion times and error rates did not differ significantly between groups. A significant correlation was observed between Super Monkey Ball scores and the Numbers module (*r* = -0.418, *p* = 0.003) as well as overall arthroscopy performance (*r* = -0.356, *p* = 0.011), indicating that higher scores were associated with shorter completion times and better performance.

**Conclusions:**

The study did not confirm that regular video gaming improves initial performance on an arthroscopic box trainer. However, the observed associations between specific video game performance measures and arthroscopic performance suggest that gaming may support the development of skills relevant to minimally invasive surgery.

**Level of Evidence:**

Level II, Prospective comparative study.

**Supplementary Information:**

The online version contains supplementary material available at https://doi.org/10.1007/s40670-026-02827-z.

## Introduction

Arthroscopy, a minimally invasive procedure, has become the gold standard for the treatment of various joint diseases and injuries [1, 2]. By utilizing smaller incisions and minimizing soft tissue disruption, arthroscopic techniques have been associated with lower rates of postoperative complications, reduced surgical morbidity, and improved short-term recovery compared to open surgical approaches [3–6]. Furthermore, this approach allows rehabilitation to be accelerated, promoting faster functional recovery and an earlier return to daily activities or work [2, 7].

Successful arthroscopic surgery requires highly specialized skills that differ from those of open surgery [8–10]. The translation of a three-dimensional operative field onto a two-dimensional screen challenges depth perception, while the fulcrum effect, where external movements produce opposite internal motions, increases the complexity of instrument handling [9, 11, 12]. The absence of direct haptic feedback further complicates performance, requiring indirect manipulation of tissues [12, 13]. Consequently, arthroscopy demands a high level of bimanual coordination, motor dexterity, and visuospatial ability [14–17].

Simulation-based training, including box trainers and virtual reality simulators, has been shown to be effective for acquiring these skills [18–20]. However, limited access, high costs, and increasing clinical workloads restrict regular training opportunities, highlighting the need for accessible adjunctive training methods [21]. In this context, video gaming has gained attention as a potential tool to enhance psychomotor and visuospatial skills relevant to minimally invasive surgery [22–24]. Several studies have investigated this relationship with heterogeneous findings. Jentzsch et al. demonstrated that experienced three-dimensional gamers performed better in arthroscopy simulation, particularly in tasks requiring triangulation, whereas two-dimensional games showed no association [25]. Similarly, Cychosz et al. reported a modest correlation between gaming experience and arthroscopic performance [26]. Furthermore, motion-controlled systems such as the Nintendo Wii have been associated with improved surgical simulator performance, an effect attributed to the kinematic similarity between controller and surgical instrument movements. However, these findings are not universal, as other studies found no significant association between gaming experience and minimally invasive surgical skills [27, 28]. A systematic review by Gupta et al. confirmed this heterogeneity, noting that while gaming appeared beneficial for laparoscopic and robotic surgery, no such benefit was consistently observed for arthroscopy, highlighting the need for further investigation in this specific domain [22].

From a theoretical perspective, the potential benefits of video gaming align with established learning theories. Many video games provide repetitive, goal-directed practice with immediate feedback, core elements of deliberate practice theory that are essential for skill acquisition [23, 29]. Furthermore, repeated engagement with complex visuomotor tasks has been shown to enhance attentional control and reduce extraneous cognitive load, facilitating more efficient technical skill learning [30, 31]. These mechanisms are particularly relevant in the context of arthroscopy, where continuous visuomotor integration under indirect visual feedback and constrained instrument maneuverability is required. Notably, such processes may not confer immediate performance advantages but rather improve the capacity to acquire novel skills more efficiently, a concept described as “learning to learn” [32, 33].

Despite these theoretical considerations and prior findings, the evidence regarding the impact of video gaming on arthroscopic performance remains inconsistent, and studies using physical box trainers are scarce. Therefore, the objective of the present study was to assess whether engagement with conventional video games influences basic arthroscopic skills using a standardized box trainer model. We hypothesized that regular video game play would be associated with improved initial performance and that video game proficiency would correlate with arthroscopic task performance.

## Methods

A total of 50 students, including 41 medical students and 9 students from other disciplines, took part in this prospective study. Participants were recruited through emails distributed via the university’s student mailing list, through semester-specific social media groups, and by direct approach. Enrollment was open to all university students regardless of discipline. Participation was voluntary and self-enrolled. The study was conducted at the Clinic of General Orthopedics and Tumor Orthopedics Department of a tertiary university hospital using an arthroscopic box trainer and two video games. After a detailed explanation of the study and its data analysis procedures, the participants gave their informed written consent. The study was approved by the local ethics committee.

The aim was to recruit 25 students who played video games for more than 3 h per week, alongside 25 students who played for less than 3 h per week or not at all. This threshold was adopted from previous studies investigating the relationship between video gaming and surgical performance [23, 34–36]. The latter group was categorized as non-gamers, while those who play more than 3 h per week were categorized as gamers. Exclusion criteria were participation in an arthroscopy course or previous practical experience with arthroscopic procedures.

As no standardized instrument for assessing video gaming experience in the context of surgical skills exists [37], a study-specific questionnaire was developed based on previously published studies in this field [24, 25, 34, 35]. The questionnaire (see Supplementary File) was completed by all participants prior to the study and covered three principal domains: (1) demographic and background data, including gender, age, handedness, educational level, field of study, and part-time medical employment [34, 35]; (2) video gaming habits, including current and childhood gaming frequency, duration of regular gaming, preferred genres, and gaming platform [24, 25]; and (3) self-perceived manual dexterity and confidence in acquiring new motor skills, assessed using Likert-scaled items [38].

**Fig. 1:**
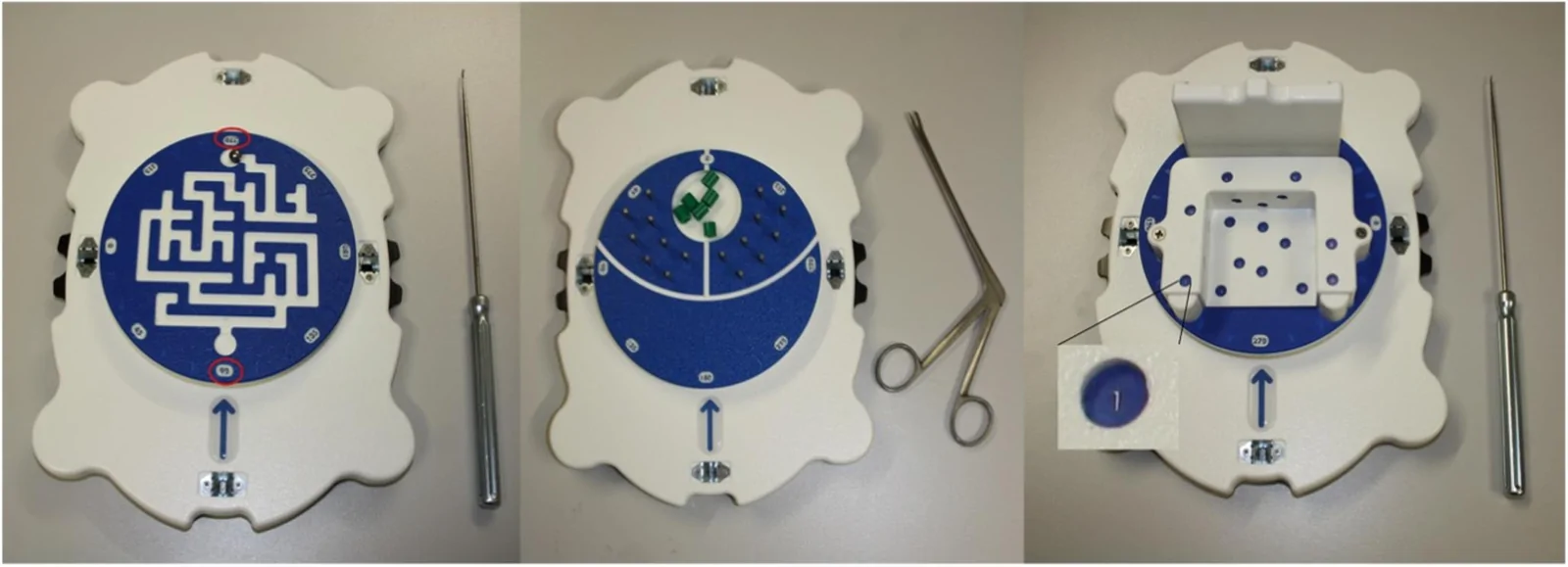
Module Maze, Module Transfer, Module Numbers (from left to right), all modules are shown without a dome

In order to eliminate any possible warm-up effect that could act as a confounding variable for subsequent tasks, participants were randomly assigned to begin with either the arthroscopy modules or the video games. The participants’ basic arthroscopic skills were assessed in three different modules using a box trainer (FAST-Trainer, Sawbones). Each module was designed to target a distinct core competency of arthroscopic surgery:

- **Module 1**,** Maze** (Fig. 1): Participants had to navigate a 7.9 mm metal ball through a maze using a standard arthroscopic probe. The ball was first positioned at number 270, and then moved to number 90, with the probe held in the dominant hand and the arthroscope in the non-dominant hand. When number 90 was reached, the instruments were swapped between the hands, and the ball was returned to position 270. This module was designed to assess triangulation and camera navigation skills, which are fundamental to all arthroscopic procedures [39, 40].
- **Module 2**,** Transfer** (Fig. 1): Participants used an arthroscopic grasper to position six cylinders in their designated locations on the module. A template with the specific positions was provided for memorization prior to the task. The cylinders were initially aligned horizontally within a white circle. The rongeur was held with the dominant hand, while the arthroscope was controlled with the non-dominant hand. This module evaluated object manipulation and grasping skills, simulating tasks such as the removal of loose bodies or tissue handling during arthroscopic procedures [40, 41].
- **Module 3**,** Numbers** (Fig. 1): Participants were asked to identify numbers from 1 to 21 in the correct order and touch them using an arthroscopic probe. After successful identification, each number was sequentially punctured with the probe. This module assessed systematic inspection and probing skills, reflecting the structured visual survey and probing required during diagnostic arthroscopy [20, 39].

The modules were carried out in a predetermined, standardized sequence for all participants to ensure comparability of results and minimize potential order effects, consistent with established practice in arthroscopic simulation studies [40, 42]. Before each module, participants received written instructions and were instructed to read them thoroughly, with the opportunity to ask questions if necessary. Once the module began, no further questions or instructions were permitted. The participants’ arthroscopic performance was documented by video recordings and subsequently assessed independently by two raters. Prior to the study, both raters were trained using a standardized protocol that included joint review of sample recordings to establish consistent application of the error categories and scoring criteria. Task performance was evaluated using a composite scoring system that incorporated time (in seconds) and predefined penalties for specific errors (Table 1). This approach follows established methodology in surgical simulation research, where composite scores combining task time with error-based deductions have been widely used for both arthroscopic and laparoscopic skills assessment [41, 43, 44]. Error categories and corresponding penalty values were defined a priori for each module and graded according to severity, with minor errors (e.g., touching module components) receiving lower penalties (1 s) and major errors (e.g., dropping objects off the module or skipping numbers) receiving higher penalties (10 s), reflecting the principle that more consequential technical errors should carry greater weight in performance assessment [45]. In cases where discrepancies in the number of errors were identified between the evaluators, the average was calculated.

**Table 1:** Error categories and corresponding time penalties for each module

Module	Error Type	Penalty per Error [seconds]
Maze	Boundary crossing	5
Maze	Module drop-off	10
Transfer	Touching module components	1
Transfer	Dropping cylinder on the plate	5
Transfer	Dropping cylinder off the plate	10
Transfer	Incorrect or missing cylinder placement	5
Numbers	Touching module components	1
Numbers	Incorrect representation	5
Numbers	Skipped number	10
Numbers	Incorrect sequence	5

To complement Table 1 and ensure transparent reproducibility, the module specific composite scores were calculated as follows:

- I. Score Maze [seconds] = Time [seconds] + Number of boundary crossings * 5 [seconds] + Number of module drop offs * 10 [seconds]
- II. Score Transfer [seconds] = Time [seconds] + Number of touches of module components * 1 [second] + Number of cylinders dropped on the plate * 5 [seconds] + Number of cylinders dropped off the plate * 10 [seconds] + Number of incorrect or missing cylinder placements * 5 [seconds]
- III. Score Numbers [seconds] = Time [seconds] + Number of touches of module components * 1 [second] + Number of incorrect representations * 5 [seconds] + Number of skipped numbers * 10 [seconds] + Number of incorrect sequences * 5 [seconds].

A total arthroscopy score was calculated by summing the scores of all three modules, and a total error count was determined across all tasks. Lower scores and lower error counts indicate better performance in arthroscopy tasks.

In order to objectively evaluate the self-reported gaming experience, the participants had to play the following two video games in a standardized order in addition to the arthroscopy tasks on the study day. The selection of games was based on prior research investigating the relationship between video gaming and surgical performance.

**Fig. 2:**
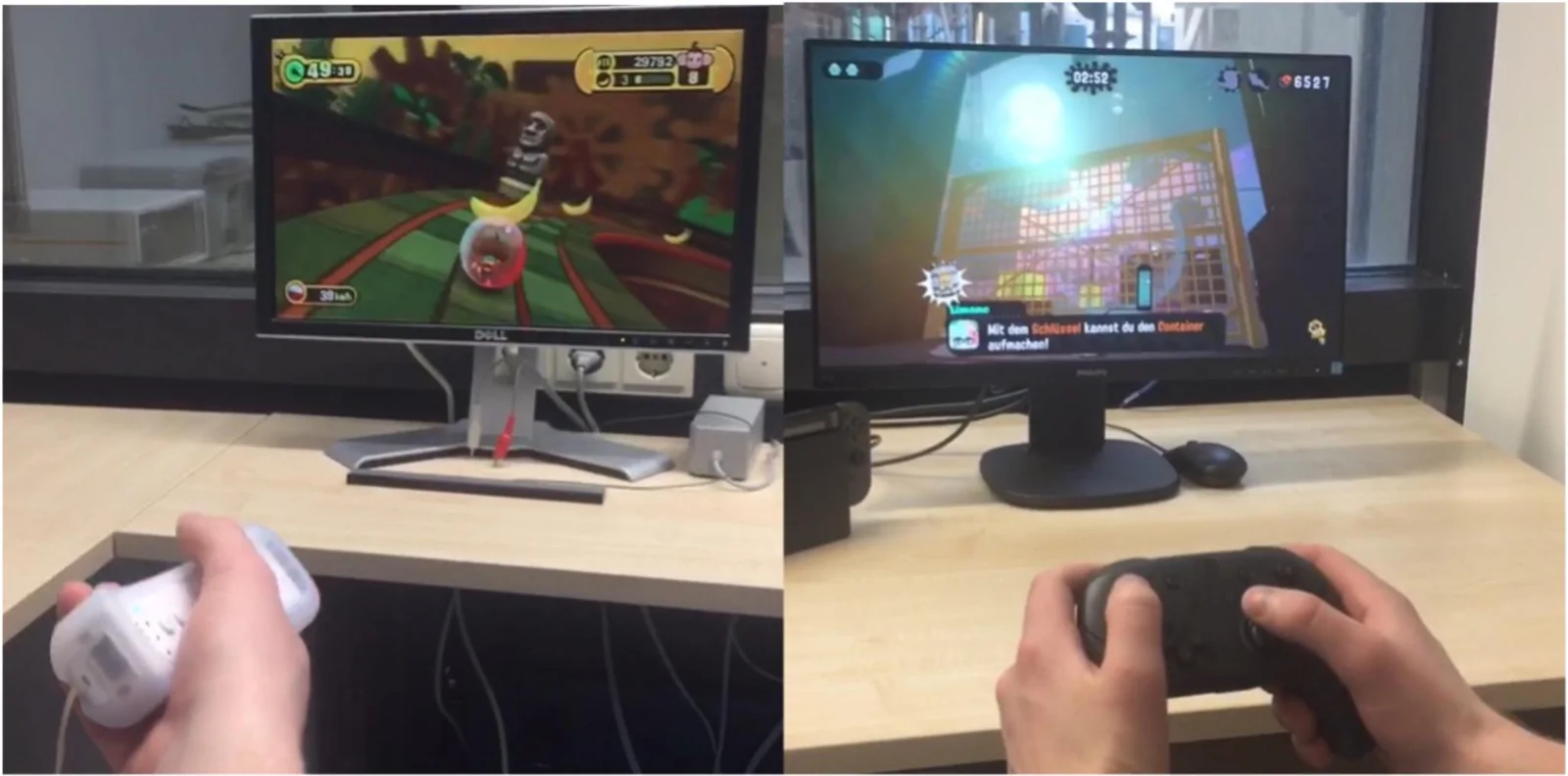
Video game Super Monkey Ball (left); video game Splatoon (right)

**Game 1**,** Super Monkey Ball Step & Roll (Sega; Ōta**,** Tokio;** Fig. 2**)**: The game was played on the Nintendo Wii using the Wii Remote Plus, which is equipped with a motion sensor and communicates wirelessly with a sensor bar placed in front of the screen, so that the character can be controlled by movement rather than by pressing buttons. The task was to guide a monkey to a specific target within a fixed time limit of 60 s, whereby 10 consecutive levels had to be completed. The monkey, enclosed in a ball, was moved by tilting the controller. Turning the controller to the right or left caused the floor to tilt in the corresponding direction and thus influenced the movement of the ball; tilting it backwards, on the other hand, reduced the speed of the ball, while tilting it forwards increased it. Super Monkey Ball was selected based on its established use in surgical simulation research, where it has demonstrated significant correlations with laparoscopic performance metrics including intracorporeal suturing [34, 46, 47].

**Game 2**,** Splatoon 2 (Nintendo; Kyōto**,** Japan;** Fig. 2**)**: The third-person shooter was played on the Nintendo Switch using the Pro Controller, which contains tilt controls additional to traditional buttons. Tilting the controller backward shifts the character’s view upward, while tilting it forward causes the character to look downward. Horizontal rotations adjust the character’s perspective, with slight shifts allowing for quick changes in viewpoint. The objective of the level used in this study was to navigate through enemies and obstacles to eventually rescue a fictional character who is being held captive. Splatoon 2 was chosen to align with prior research demonstrating strong correlations between three-dimensional shooter games and arthroscopic simulator performance [25], as well as evidence that visually complex shooter games enhance visuospatial skills relevant to endoscopic surgery [48].

A composite score was calculated for each video game to provide an objective measure of in-game performance. The scores incorporated each game’s native performance metrics, including points achieved, lives remaining, and time efficiency. This approach was adopted in line with prior studies that used objective in-game performance data to quantify gaming proficiency [24, 25, 34]. As no standardized scoring system for video game assessment in surgical research exists [37], the composite formulas were designed to capture overall game performance in a single quantifiable metric. The weighting of individual components was defined a priori to reflect their relative contribution to overall game performance, with higher weights assigned to task-relevant achievements such as level progression and objective completion. The module-specific composite scores were calculated as follows:

- IV. Score Super Monkey Ball = In-game score+ (Number of remaining lives* 20) – (Number of lost lives* 20).
- V. Score Splatoon= (Number of checkpoints reached* 1000) + (Number of lives earned * 10) – (Number of lives lost* 10) + Points – Time [seconds]/Checkpoints * 100.

In contrast to the arthroscopy scores where lower values indicate better performance, higher composite scores reflect better performance in the video game tasks.

### Statistics

Data collection was conducted using Excel 2013 (Microsoft, Redmond, WA, USA), with statistical analysis and graphical representations performed using IBM SPSS Statistics (IBM, Armonk, NY, USA; Version 25.0) and RStudio (Posit Software, Boston, MA). Normal distribution was evaluated using histograms, skewness, and the Shapiro-Wilk test. For group comparisons, the t-test was applied to normally distributed variables and the Mann-Whitney U test to non-normally distributed variables. Pearson’s correlation coefficient was used to assess bivariate associations between normally distributed continuous variables. Interrater reliability was assessed using intraclass correlation coefficients (ICC, two-way random effects model, absolute agreement).

To identify independent predictors of total arthroscopy performance, univariate and multivariable linear regression analyses were performed. The variables included in the regression models (age, sex, handedness, weekly gaming hours, childhood gaming experience, and self-perceived dexterity) were selected based on factors previously reported to influence performance in surgical simulation studies, including gaming experience, demographic characteristics, and self-assessed manual ability [27, 28, 34]. Given the sample size of 50 participants, the number of predictor variables was limited to avoid model overfitting. All variables were entered simultaneously into the multivariable model. Statistical significance was set at *p* < 0.05.

## Results

### Study Group

The study comprised 50 participants, including 41 medical students and 9 individuals from other disciplines, from semesters 1 through 10. Among the participants, 38 (76%) were male and 12 (24%) female. The mean age was 23.04 years (SD ± 2.4), ranging from 19 to 30 years. In terms of handedness, 42 (84%) were right-handed and 8 (16%) left-handed.

No significant differences were observed between the gamer and non-gamer groups in terms of gender distribution, age (*p* = 0.294), or handedness (*p* = 0.702). The groups differed significantly in the hours played per week and years of regular gameplay (*p* < 0.001 for both). Detailed information on gaming habits and preferred game genres, stratified by group, is provided in Supplementary Table 1. Additional questionnaire data, including gaming experience, self-perceived dexterity, and engagement in dexterity-related activities, are summarized in Supplementary Table 2.

### Interrater Reliability

Interrater reliability for the assessment of arthroscopic performance was excellent across all three modules. The ICC values were 0.982 for the Maze module (95% CI, 0.967–0.990), 0.985 for the Transfer module (95% CI, 0.974–0.991), and 0.922 for the Numbers module (95% CI, 0.866–0.955).

### Comparison of Game Performance

**Fig. 3:**
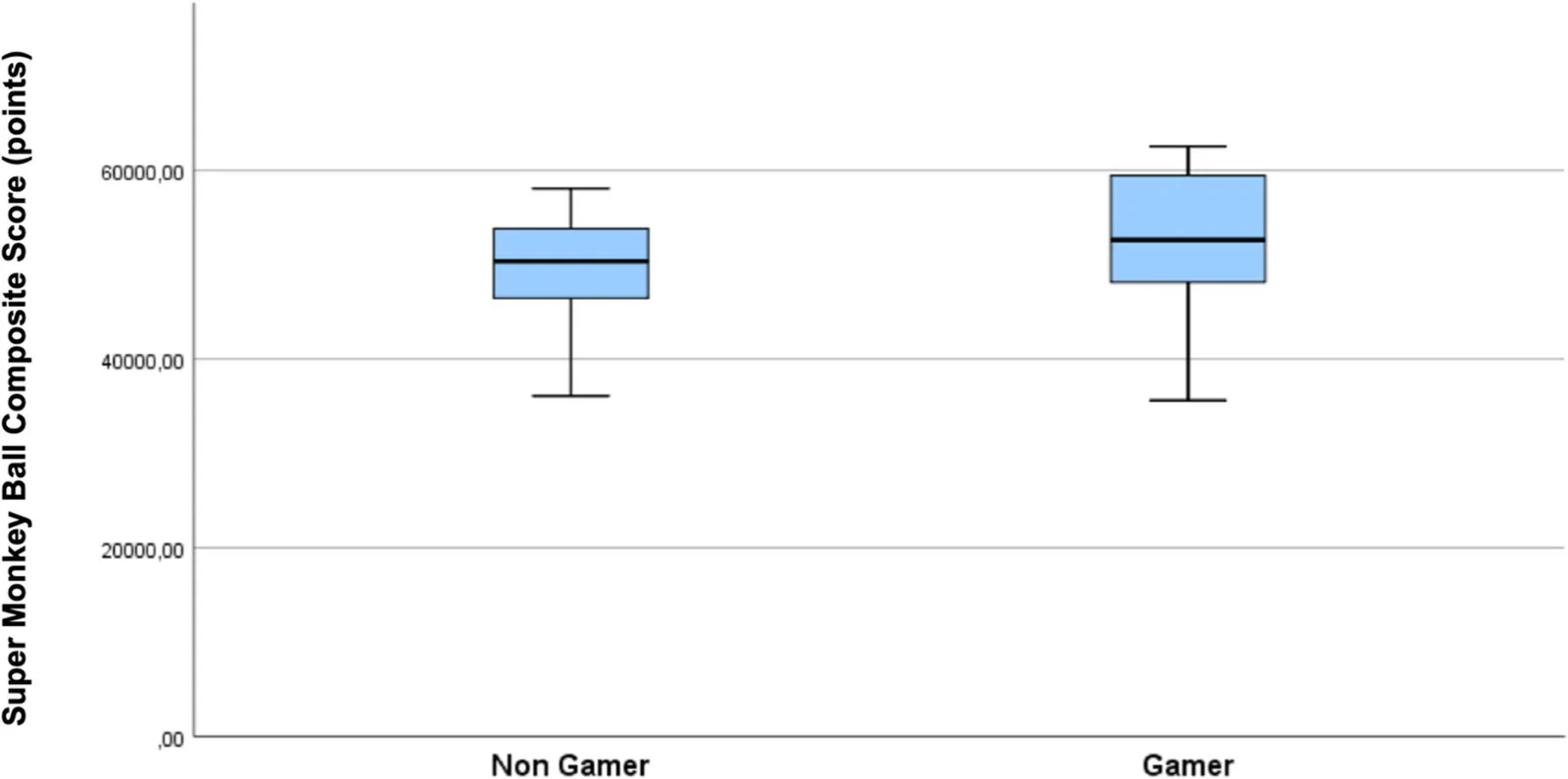
Boxplots illustrating Super Monkey Ball composite scores in the non-gamer and gamer groups. The central line represents the median, boxes indicate the interquartile range (IQR), and whiskers represent the range of non-outlier values. The difference between the groups was not statistically significant (*p* = 0.172)

**Fig. 4:**
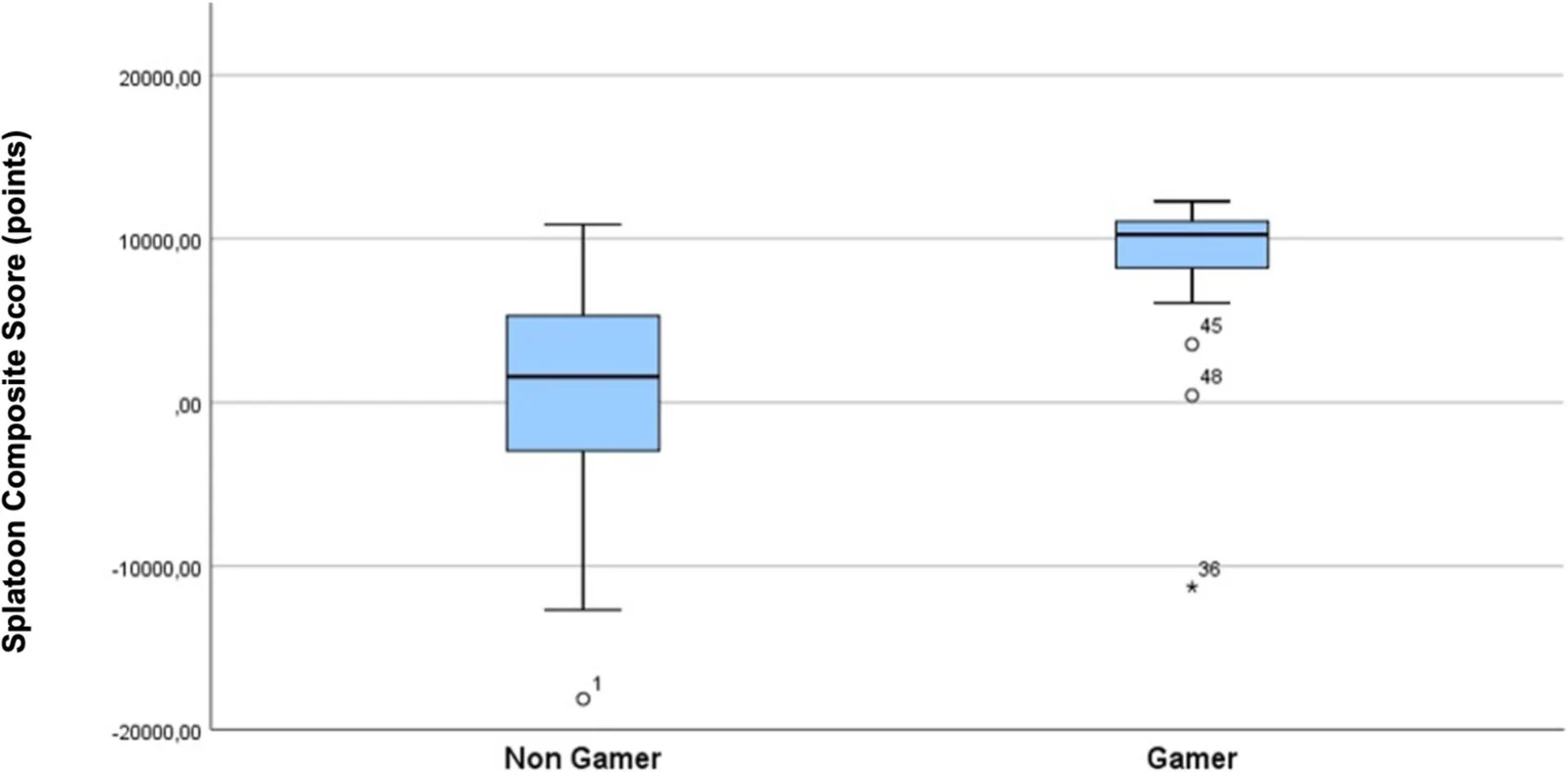
Boxplots illustrating Splatoon composite scores in the non-gamer and gamer groups. The central line represents the median, boxes indicate the interquartile range (IQR), and whiskers represent the range of non-outlier values. The numbers displayed next to the circles and asterisks indicate the case numbers of mild and extreme outliers identified during the analysis. Gamers achieved substantially higher composite scores compared to non-gamers (*p* < 0.001)

In Super Monkey Ball, non-gamers achieved a mean score of 49,864 points, compared to 52,543 points for gamers. The discrepancy of 2,679 points was not statistically significant (*p* = 0.172; Fig. 3). In Splatoon, non-gamers achieved an average of 616 points, while gamers achieved 8,581 points, indicating a statistically significant difference (*p* < 0.001; Fig. 4).

#### Comparison of Arthroscopy Modules

Composite scores in the Maze (*p* = 0.432), Transfer (*p* = 0.426), and Numbers modules (*p* = 0.227) did not differ significantly between gamers and non-gamers. The overall arthroscopy score also showed no significant difference between groups (*p* = 0.566). Similarly, no statistically significant differences in error counts were identified between the two groups for any module (all *p* > 0.2). A summary of descriptive statistics for arthroscopy performance by group is presented in Table 2.

**Table 2:** Descriptive statistics for arthroscopy module performance, stratified by group. Values are presented as mean ± standard deviation. Composite scores incorporate task completion time and predefined error-based penalties, with lower scores indicating better performance

Variable	Non-Gamers (Mean ± SD)	Gamers (Mean ± SD)	*P*-value
Maze score	392.9 ± 114.5	442.5 ± 173.3	0.432
Maze errors	2.3 ± 2.0	1.7 ± 1.8	0.241
Transfer score	476.4 ± 241.6	416.6 ± 180.6	0.426
Transfer errors	13.1 ± 9.4	11.3 ± 6.3	0.808
Numbers score	828.8 ± 201.6	772.7 ± 103.7	0.227
Numbers errors	10.4 ± 5.6	10.2 ± 3.9	0.580
Total score	1698.1 ± 464.6	1631.9 ± 334.0	0.566
Total errors	25.8 ± 14.1	23.2 ± 8.3	0.969

### Correlation of Game Scores with Arthroscopy Performance

**Fig. 5:**
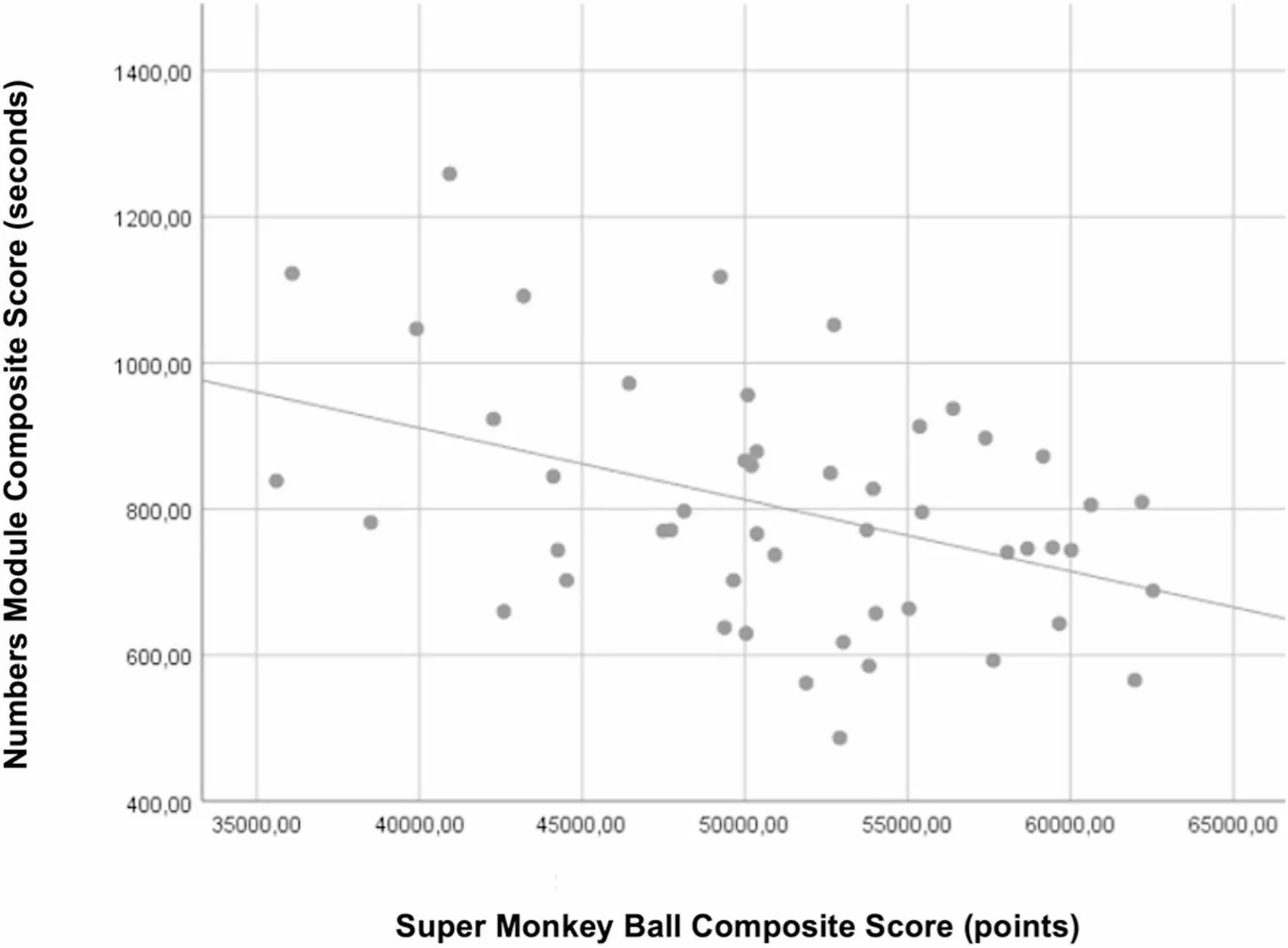
Scatter plot illustrating the correlation between the Numbers module composite score and the Super Monkey Ball composite score. The Pearson correlation was *r* = − 0.418 and reached statistical significance (*p* = 0.003). Lower scores in the Numbers module indicate better performance in the arthroscopy task, while higher scores in Super Monkey Ball reflect better performance in the video game. The negative correlation therefore shows that participants who performed better in the video game tended to achieve better results in the Numbers module

**Fig. 6:**
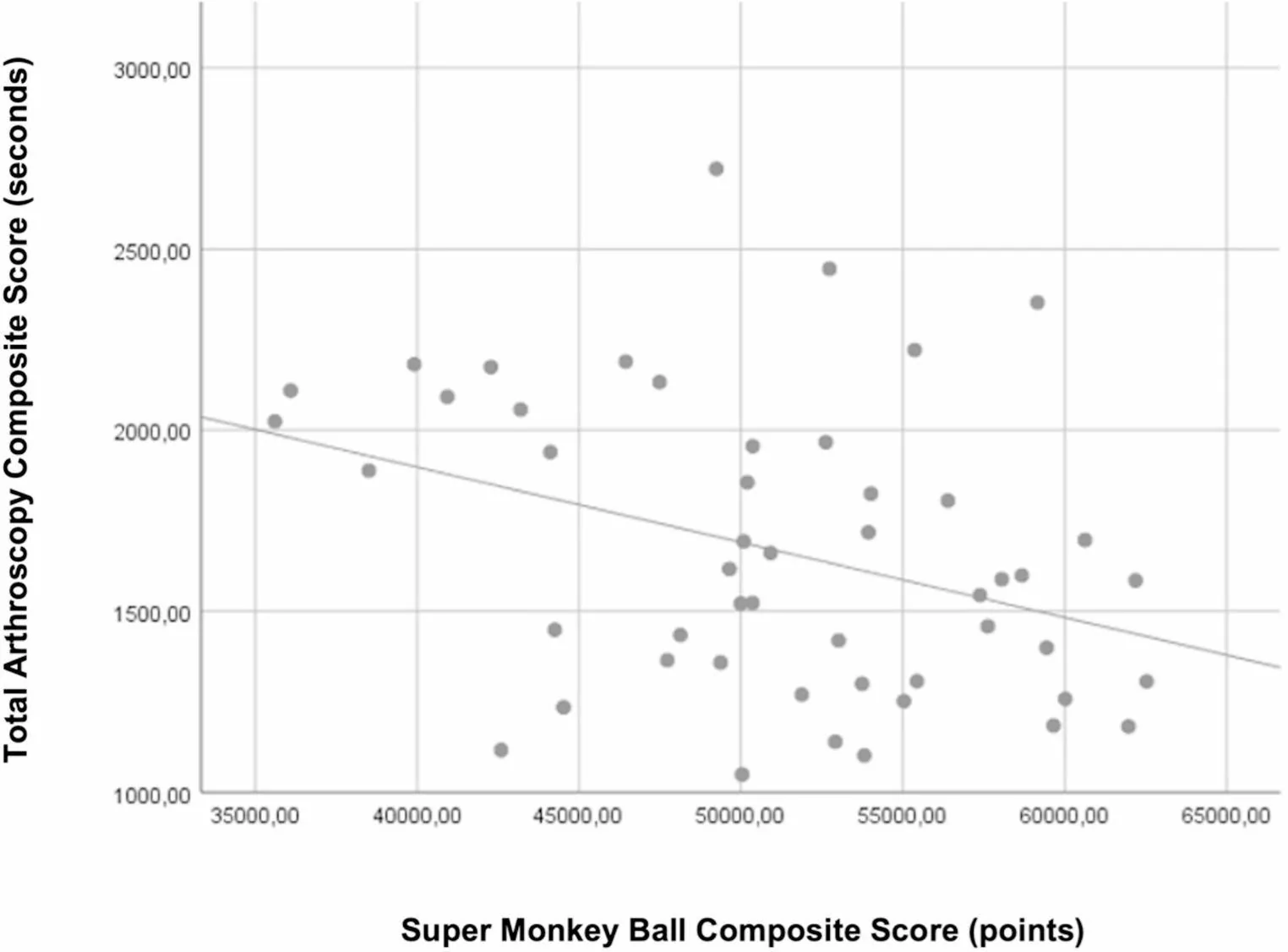
Scatter plot illustrating the correlation between the total arthroscopy composite score and the Super Monkey Ball composite score. The Pearson correlation was *r* = − 0.356 and reached statistical significance (*p* = 0.011). Lower total arthroscopy scores indicate better overall performance in the arthroscopy tasks, while higher Super Monkey Ball scores reflect better performance in the video game. The negative correlation therefore shows that participants who performed better in Super Monkey Ball tended to achieve better overall results in the arthroscopy assessment

A significant Pearson correlation was identified between the Super Monkey Ball score and the Numbers module score (*r* = -0.418, *p* = 0.003; Fig. 5), as well as the overall arthroscopy score (*r* = -0.356, *p* = 0.011; Fig. 6). Since lower arthroscopy scores indicate better task performance and higher Super Monkey Ball scores indicate better game performance, the negative coefficients reflect that higher Super Monkey Ball performance was associated with better performance in the Numbers module and in the overall arthroscopy score. However, no statistically significant correlations were revealed between the Super Monkey Ball score and the Maze (*r* = -0.241, *p* = 0.092) or Transfer (*r* = -0.186, *p* = 0.197) modules.

Furthermore, no significant correlations were found between the Super Monkey Ball score and the number of errors in any module. Similarly, no statistically significant correlations were detected between the Splatoon scores and the arthroscopy module scores or errors.

#### Influence of Factors on Arthroscopy Performance

In univariate analyses, self-perceived dexterity was the only variable significantly associated with the total arthroscopy score (β = 234.0, 95% CI 102.0–366.0, *p* < 0.001), while weekly gaming hours, age, sex, handedness, and childhood gaming experience showed no significant associations (Supplementary Table 3). This finding was confirmed in the multivariable linear regression model (Table 3), in which self-perceived dexterity remained the only independent predictor of total arthroscopy performance (β = 210.67, 95% CI 75.0–346.0, *p* = 0.003).

**Table 3:** Multivariable linear regression analysis of predictors of total arthroscopy composite score. All variables were entered simultaneously into a single model. Positive β coefficients indicate higher (i.e., worse) arthroscopy scores. Self-perceived dexterity was assessed on a 5-point Likert scale (1 = applies, 5 = does not apply), with higher values reflecting lower self-rated dexterity. Significant values (*p* < 0.05) are bolded

Variable	Estimate (ß)	95% confidence interval		*P*-value
		Lower	Upper	
Age	30.28	-12.6	73.1	0.161
Sex [Female]	234.68	-30.1	499.0	0.081
Handedness [Left]	275.91	-7.12	559.0	0.056
Weekly gaming hours	-6.95	-20.3	6.43	0.301
Childhood gaming [No]	-91.69	-339.0	155.0	0.458
Self-perceived dexterity	210.67	75.0	346.0	**0.003**

## Discussion

This study examined the association between video gaming and arthroscopic performance in 50 participants. Gamers achieved significantly higher scores in Splatoon, while no group differences were observed in Super Monkey Ball. In the arthroscopy modules, composite scores and error counts, and overall performance did not differ significantly between gamers and non-gamers. Importantly, higher scores in Super Monkey Ball were associated with better composite scores in the Numbers module as well as in the overall arthroscopy assessment.

Although the relationship between video gaming and minimally invasive surgical skills has been studied extensively in laparoscopy, evidence in arthroscopy remains limited [35, 49, 50]. Addressing this gap, Jentzsch et al. investigated the association between video game experience and arthroscopy simulator performance by examining 30 volunteers without prior arthroscopy experience. Participants completed three tasks on a validated virtual reality knee arthroscopy simulator and were assessed for video game experience using both a questionnaire and actual performance in five two- and three-dimensional video games across two platforms. The study observed correlations between arthroscopy simulator performance and both video game performance and experience. The authors concluded that skilled three-dimensional gamers performed better in arthroscopy simulation than inexperienced gamers [25]. Cychosz et al. also identified a correlation between video game use and arthroscopy performance, although this association was weak. In their study, 43 medical students without prior arthroscopy experience performed a diagnostic knee arthroscopy on a virtual reality simulator. Baseline performance correlated only modestly with current and previous gaming, whereas performance gains during repeated training were more strongly associated with prior participation in sports activities requiring hand-eye coordination [26]. Although the aforementioned studies suggest a correlation between video game performance or experience and improved outcomes in arthroscopic tasks, the overall evidence remains heterogeneous. Richey et al. evaluated 49 medical students using a knee arthroscopy simulator to investigate the influence of various lifestyle factors, including athletic participation, musical training, and video gaming. Their analysis demonstrated that high levels of athletic experience were significantly associated with superior simulator performance and a higher probability of achieving competency, whereas video gaming experience showed no significant relationship with arthroscopic skills [28]. Likewise, Rahm et al. reported no association between video game experience and improved arthroscopy performance. In their prospective study, 20 medical students completed eight sessions of structured training on a validated knee arthroscopy virtual reality simulator. Baseline abilities were assessed using visuospatial tests, questionnaires on prior gaming experience, and other potential predictors of technical skill. The authors reported no significant influence of video game experience, visuospatial ability, or other measured factors on initial performance or subsequent learning curves, concluding that no consistent predictors of natural aptitude for arthroscopy could be identified [27].

The findings of the present study align with the aforementioned conflicting evidence. Contrary to our first hypothesis, regular video gaming did not translate into superior initial performance on the arthroscopic box trainer, as no differences were observed between gamers and non-gamers. In contrast, our second hypothesis was partly supported, as performance in Super Monkey Ball showed a significant association with both the Numbers module and overall arthroscopy performance, indicating that higher game scores corresponded to better composite scores. These results suggest that measurable proficiency in specific video games may be a more relevant indicator of arthroscopic ability than gamer status based on self-reported weekly playtime. The divergent results between Super Monkey Ball and Splatoon may also be related to the choice of console. Importantly, the two games were deliberately selected to represent distinct gameplay mechanics, with Super Monkey Ball relying on motion-based controller input and fine motor coordination, whereas Splatoon primarily involves three-dimensional visuospatial processing and rapid target acquisition. Prior research in laparoscopy has shown that games played on the Nintendo Wii associated strongly with the development of minimally invasive surgical skills. Giannotti et al. demonstrated that students without prior laparoscopic or gaming experience performed significantly better on a simulator after four weeks of Wii-based training compared to controls [51]. Ju et al. further compared the Wii with the PlayStation 2 and reported positive effects for both systems, with slightly greater improvements for the Wii [52]. Likewise, Kulkarni et al. found stronger correlations between laparoscopic skills and Wii performance compared to the Xbox, attributing this to the similarity between Wii controller movements and surgical instrument handling [53]. These findings may explain why our study identified a significant association for Super Monkey Ball on the Wii but not for Splatoon on the Switch.

Notably, multivariable regression analysis identified self-perceived dexterity as the only independent predictor of total arthroscopy performance, while weekly gaming hours, age, sex, handedness, and childhood gaming experience showed no significant associations. This finding is consistent with prior research suggesting that self-assessed manual ability may reflect underlying visuomotor confidence relevant to surgical simulation performance. Gangemi et al. reported that individuals who rated their manual dexterity and motor coordination as high performed significantly better on surgical dexterity tasks [38].

Beyond these findings, several strengths of the present study should be highlighted. This investigation is, to our knowledge, one of the few studies to examine arthroscopic performance using a physical box trainer rather than an exclusively virtual reality based system. This approach provides a more tangible representation of instrument handling, triangulation, and depth perception, thereby creating more realistic training conditions and allowing conclusions that are more applicable to real world arthroscopy training. Furthermore, the study design combined subjective measures of gaming experience with objective in game performance metrics, which enabled a more nuanced analysis of how video gaming relates to technical skills. Notably, the observation that measurable performance in Super Monkey Ball correlated with both the Numbers module and the overall arthroscopy assessment highlights the potential value of selected video games as accessible and low cost adjuncts for the early identification of visuomotor aptitude. From a clinical and educational perspective, these results may contribute to the development of targeted preparatory or supplementary training tools for novice learners. If certain motion based games reliably reflect aspects of arthroscopic skill, they could be integrated into preclinical training curricula or used to support individualized learning pathways by identifying students who may benefit from additional visuospatial skill development before entering simulator based or clinical training.

We acknowledge several limitations to our study. Firstly, gamers did not perform significantly better than non-gamers in Super Monkey Ball, despite reporting at least three hours of weekly gaming. This questions the adequacy of our gamer classification and may account for the absence of significant differences in arthroscopy performance between the groups. Secondly, although the sample size of 50 students is larger than many comparable studies, it is still relatively small. A larger sample would increase the significance of the results. Thirdly, due to the voluntary nature of participation, there is a possibility that students with a particular interest in arthroscopic procedures were disproportionately represented in the cohort, which could lead to selection bias. Fourthly, due to the absence of a standardized instrument for assessing gaming experience in the context of surgical skills [37], a study-specific questionnaire was developed, which has not undergone formal external validation. However, its content domains were informed by previously published studies in this field [25, 34, 35].

## Conclusion

The hypothesis that regular engagement with video games is associated with improved initial performance on an arthroscopic box trainer was not confirmed, as no significant differences were observed between gamers and non-gamers. Nevertheless, significant correlations were observed between performance in Super Monkey Ball and arthroscopic outcomes, both in individual modules and in the overall assessment. These findings suggest that objectively measured proficiency in specific motion-controlled video games may reflect visuomotor abilities relevant to arthroscopic surgery, though no causal relationship can be inferred. Selected video games may serve as a potential low-cost adjunct for early arthroscopy training and for identifying learners with strong baseline technical aptitude. Further research with larger cohorts is required to confirm these findings. In addition, greater standardization in the selection of video games across studies would improve comparability within this field of research. The inclusion of participants at different stages of surgical training would help to determine whether the observed associations translate into measurable benefits in clinical arthroscopic performance.

## Supplementary Information

Below is the link to the electronic supplementary material.

Supplementary Material 1

## Data Availability

The datasets generated and analyzed during the current study are available from the corresponding author upon reasonable request.
