# Supplementary material for: Influence of Regular Video Game Play on the Basic Skills of Arthroscopic Surgery – Results of a Prospective Study
Source: Med Sci Educ. 2026 Jul 20;36(5):2201–12. doi: 10.1007/s40670-026-02827-z (PMC13723654; doi:10.1007/s40670-026-02827-z)
Supplement: Supplementary file 1 [file 40670_2026_2827_MOESM1_ESM.docx]

**SUPPLEMENTARY DATA:**

***Supplementary Table 1.*** *Self-reported weekly gaming hours by genre, stratified by group. Values are presented as mean hours per week based on participant self-report. MOBA = Multiplayer Online Battle Arena.*

| **Genre** | **Non-Gamers (hours/week, mean)** | **Gamers (hours/week, mean)** |
| --- | --- | --- |
| First-person shooter | 0.00 | 4.70 |
| Sports games | 0.08 | 0.24 |
| Platform (Jump and Run) | 0.00 | 0.40 |
| Racing games | 0.00 | 0.34 |
| Role-playing games | 0.00 | 2.40 |
| MOBA | 0.04 | 1.16 |
| Strategy | 0.04 | 1.02 |
| Puzzle games | 0.10 | 0.82 |
| Other | 0.00 | 1.24 |
| Total | 0.26 | 12.32 |

***Supplementary Table 2.*** *Questionnaire responses by group. Values are presented as mean ± SD, median (IQR), or n (%) as appropriate. Likert-scaled items were rated on a 5-point scale (1 = applies, 5 = does not apply), with lower values indicating stronger agreement. DIY = Do-It-Yourself. Significant values (p < 0.05) are shown in bold.*

| **Variable** | **Non-Gamers (n=25)** | **Gamers (n=25)** | **P-value** |
| --- | --- | --- | --- |
| ***Medical background*** | | | |
| **Part-time medical employment, n (%)** | **15 (60%)** | **4 (16%)** | **0.003** |
| ***Gaming experience*** | | | |
| **Ever used a controller, n (%)** | **23 (92%)** | **25 (100%)** | **0.490** |
| **Console ownership, n (%)** | **6 (24%)** | **24 (96%)** | **< 0.001** |
| **Childhood gaming, n (%)** | **12 (48%)** | **21 (84%)** | **0.016** |
| **Years of regular gaming, mean ± SD** | **0.76 ± 2.0** | **12.1 ± 5.3** | **< 0.001** |
| **Weekly gaming hours, mean ± SD** | **0.26 ± 0.48** | **12.3 ± 8.4** | **< 0.001** |
| ***Self-perceived dexterity and confidence (Likert 1–5), median (IQR)*** | | | |
| **I consider myself skillful** | **3 (2–3)** | **2 (2–3)** | **0.770** |
| **Ease of learning new manual tasks** | **3 (2–3)** | **2 (2–3)** | **0.306** |
| **Expected ease of study tasks** | **3 (3–3)** | **3 (2–3)** | **0.116** |
| **Ease of learning new video games** | **3 (2–4)** | **2 (1–2)** | **<0.001** |
| **Interest in arthroscopic skills** | **1 (1–2)** | **1 (1–2)** | **0.480** |
| ***Regular engagement in dexterity-related activities (Likert 1–5), median (IQR)*** | | | |
| **Musical instrument** | **4 (2–5)** | **5 (4–5)** | **0.057** |
| **Handicrafts** | **4.5 (3.75–5)** | **5 (4–5)** | **0.486** |
| **Dexterity-based games** | **5 (4–5)** | **3 (2–5)** | **0.003** |
| **Manual work/DIY** | **3 (2–4)** | **2 (2–4)** | **0.702** |

***Supplementary Table 3.*** *Univariate linear regression analyses of potential predictors of total arthroscopy composite score. Each variable was analyzed in a separate model. Positive β coefficients indicate higher (i.e., worse) arthroscopy scores. Self-perceived dexterity was assessed on a 5-point Likert scale (1 = applies, 5 = does not apply), with higher values reflecting lower self-rated dexterity.* *Significant values (p<0.05) are bolded.*

| **Variable** | **Estimate (ß)** | **95% confidence interval** | | **P-value** |
| --- | --- | --- | --- | --- |
|  |  | **Lower** | **Upper** |  |
| Age | 17.80 | -30.2 | 65.8 | 0.459 |
| Sex [Female] | 236.00 | -26.0 | 497.0 | 0.076 |
| Handedness [Left] | 206.00 | -103.0 | 515.0 | 0.187 |
| Weekly gaming hours | -7.08 | -20.7 | 6.57 | 0.302 |
| Childhood gaming [No] | 30.10 | -213.0 | 274.0 | 0.805 |
| Self-perceived dexterity | 234.00 | 102.0 | 366.0 | **<0.001** |
